# Mogrol stimulates G-protein-coupled bile acid receptor 1 (GPBAR1/TGR5) and insulin secretion from pancreatic β-cells and alleviates hyperglycemia in mice

**DOI:** 10.1038/s41598-024-53380-x

**Published:** 2024-02-08

**Authors:** Chisato Tanaka, Naoki Harada, Yoshiaki Teraoka, Hiroki Urushizaki, Yoh Shinmori, Teruaki Onishi, Yusuke Yotsumoto, Yuta Ito, Tomoya Kitakaze, Takashi Inui, Yuji Murata, Hiroshi Inui, Ryoichi Yamaji

**Affiliations:** 1 Division of Applied Life Sciences, Graduate School of Life and Environmental Sciences, Osaka Prefecture University, Sakai, Osaka Japan; 2 Department of Applied Biological Chemistry, Graduate School of Agriculture, Osaka Metropolitan University, 1-1 Gakuencho, Naka-ku, Sakai, Osaka 599-8531 Japan; 3 Natural Materials Laboratory, Saraya Company, Ltd., 24-12 Tamatecho, Kashiwara, 582-0028 Kashiwara, Osaka Japan; 4 Department of Health and Nutrition, Otemae University, Osaka, Osaka Japan; 5 Center for Research and Development of Bioresources, Osaka Metropolitan University, Sakai, Osaka Japan

**Keywords:** Biochemistry, Diseases, Endocrinology, Health care

## Abstract

Target identification is a crucial step in elucidating the mechanisms by which functional food components exert their functions. Here, we identified the G-protein-coupled bile acid receptor 1 (GPBAR1, also known as TGR5) as a target of the triterpenoid mogrol, a class of aglycone mogroside derivative from *Siraitia grosvenorii*. Mogrol, but not mogrosides, activated cAMP-response element-mediated transcription in a TGR5-dependent manner. Additionally, mogrol selectively activated TGR5 but not the other bile acid-responsive receptors (i.e., farnesoid X receptor, vitamin D receptor, or muscarinic acetylcholine receptor M3). Several amino acids in TGR5 (L71A^2.60^, W75A^ECL1^, Q77A^ECL1^, R80A^ECL1^, Y89A^3.29^, F161A^ECL2^, L166A^5.39^, Y240A^6.51^, S247A^6.58^, Y251A^6.62^, L262A^7.35^, and L266A^7.39^) were found to be important for mogrol-induced activation. Mogrol activated insulin secretion under low-glucose conditions in INS-1 pancreatic β-cells, which can be inhibited by a TGR5 inhibitor. Similar effects of mogrol on insulin secretion were observed in the isolated mouse islets. Mogrol administration partially but significantly alleviated hyperglycemia in KKAy diabetic mice by increasing the insulin levels without affecting the β-cell mass or pancreatic insulin content. These results suggest that mogrol stimulates insulin secretion and alleviates hyperglycemia by acting as a TGR5 agonist.

## Introduction

Mogrol is a triterpene derived from *Siraitia grosvenorii*, used in traditional Chinese medicine. In this *Cucurbitaceae* plant, mogrol exists primarily in the form of glycosides called mogrosides that have been widely used as a natural sweetener. Mogroside V is a penta-glucose-bound mogroside that is the most potent sweetening agent in this plant. After intake of mogrosides, mogrol is produced by deglycosylation of mogrosides by gastrointestinal enzymes and intestinal microbiota. Mogrol and some mogrosides can be absorbed into the body^1,2^. Mogrol exerts several biological activities, including ameliorating inflammation in the gut^3^ and the brain^4,5^, attenuating osteoclast formation^6^, and stimulating preadipocyte differentiation^7^. To date,  the effects of mogrol on hyperglycemia and the molecular targets through which mogrol exerts its functions have not been elucidated.

G-protein-coupled bile acid receptor 1 (GPBAR1, also referred to as Takeda G-protein receptor 5 (TGR5)) acts as a bile acid receptor. TGR5 activates Gαs subunits of trimeric G-proteins^8^, inducing cAMP production and leading to transcription of cAMP-response element (CRE)-regulated genes. Bile acids stimulate gene transcription by activating the nuclear receptor, farnesoid X receptor (FXR)^9^ in addition to TGR5. Vitamin D receptor (VDR)^10^ and muscarinic acetylcholine receptor M3 (CHRM3)^11^ can also be a target of several bile acids^12^. In addition to synthetic steroidal (*e.g.*, INT-777) and synthetic non-steroidal agonists, recent studies have shown that the terpenoid compounds betulinic acid^13,14^, oleanolic acid^15,16^, maslinic acid^17^, quinovic acid^18^, and ursolic acid^19^ can activate TGR5. The limonoids (highly oxygenated terpenoids) nomilin and obacunone, the terpene lactone farnesiferol, and the terpenoid saponin glycyrrhizic acid also act as TGR5 agonists^20–23^. However, how terpenoids bind to and activate TGR5 and which physiological functions are involved in this activation remain poorly understood. Targeting GPCRs with functional food factors has recently become topic of interest^24–28^.

Type 2 diabetes mellitus (T2DM) is a major public health concern worldwide. Compared to that in 2021, the prevalence of diabetes is estimated to increase by approximately 1.5-fold, affecting more than 1.3 billion people, by 2050^29^. Excessive sugar intake is associated with T2DM^30,31^, highlighting the need for sweeteners to curb the sugar intake. In addition, functional ingredients suppressing the blood glucose levels are valuable for the prevention of hyperglycemia. Insufficient insulin release from pancreatic β-cells triggers hyperglycemia. TGR5 is expressed in pancreatic β-cells, and TGR5 activation stimulates insulin secretion^16^. Compounds that activate β-cell TGR5 are thus expected to suppress elevated blood glucose concentrations. Because mogrol and bile acids display structural similarities, we evaluated whether mogrol can activate TGR5 and found that it does. We subsequently examined whether this activation is associated with physiological functions of mogrol in diabetic animal model.

## Results

### Mogrol activated TGR5 but did not activate other bile acid receptors

**Figure 1:**
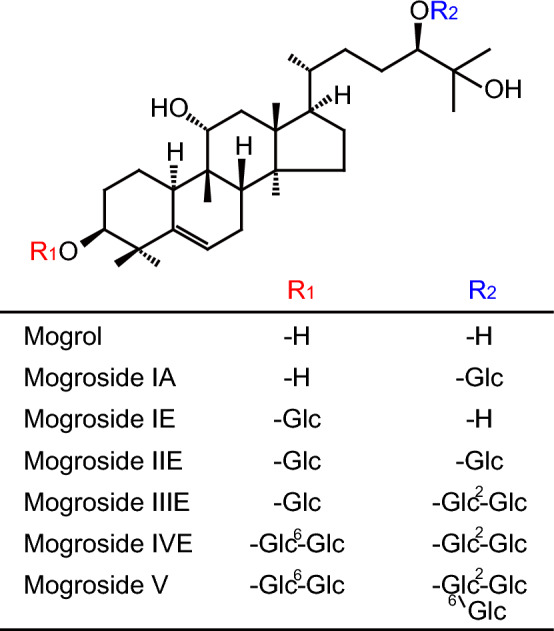
Mogrol and mogrosides structures. Glc represents the β-d-glucopyranosyl group.

**Figure 2:**
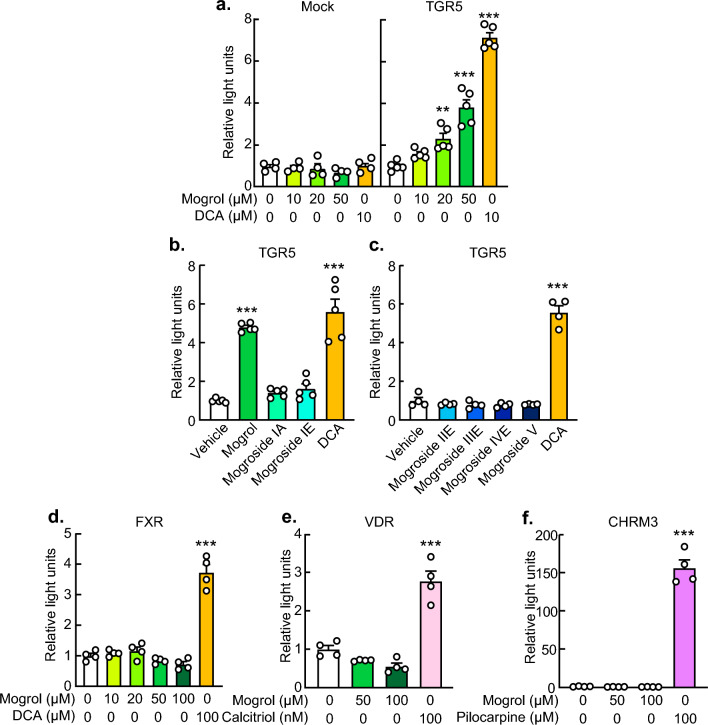
Mogrol, but not mogrosides, selectively activated TGR5 activity but not other bile acid receptors. (**a**–**f**) HEK293FT cells were transiently transfected with a receptor expression vector ((**a**–**c**) pcDNA3.2-TGR5, (**d**) p3xFlag-FXR, (**e**) pcDNA3.2-VDR, and (**f**) pcDNA3.2-CHRM3), a firefly luciferase reporter vector ((**a**–**c**) p4xCRE-TATA-Luc2P, (**d**) p4xFXRE-TATA-Luc2P, (**e**) p2xVDRE-TATA-Luc2P, and (**f**) p9xNFAT-RE-TATA-Luc2P) and a *Renilla* luciferase reporter vector pGL4.74[*hRluc*/TK] for 24 h. After stimulation with the indicated concentrations of mogrol, 50 μM mogrosides, 10 or 100 μM sodium deoxycholate (for TGR5 or FXR), 100 nM calcitriol, or 100 μM pilocarpine hydrochloride for 4 h, luciferase reporter activities were determined. Data are expressed as means ± SEM (n = 4–5). Asterisks indicate statistically significant differences compared with relevant controls (one-way analysis of variance with Dunnett’s post hoc tests, *p < 0.05, **p < 0.01, ***p < 0.001). CHRM3, cholinergic receptor muscarinic 3; FXR, farnesoid X receptor; TGR5, G-protein-coupled bile acid receptor 1/Takeda G-protein receptor 5; VDR, vitamin D receptor.

The structures of mogrosides from *S. grosvenorii* and their aglycone, mogrol, are shown in Fig. 1. TGR5 couples to Gαs^8^ and its activation is determined by using luciferase reporter assay with CRE^32,33^. Mogrol dose-dependently induced CRE-mediated transcriptional activity at concentrations over 20 μM in cells overexpressing TGR5 (Fig. 2a, p < 0.01 at 20 μM, *p* < 0.001 at 50 μM). Mogrol at 10 μM was also significant when compared to control with Student’s *t*-test (*p* = 0.003). By contrast, mogrosides IA, IE, IIE, IIIE, IVE, and V did not activate TGR5 at 50 μM (Fig. 2b and c). Unlike deoxycholate (DCA), mogrol did not activate FXR at concentrations up to 100 μM (Fig. 2d). Neither the vitamin D receptor (VDR) nor the muscarinic acetycholine receptor M3 (CHRM3), which have been reported to be targets of bile acids, were activated by mogrol (Fig. 2e and f).

### Simulated docking of mogrol to TGR5

**Figure 3:**
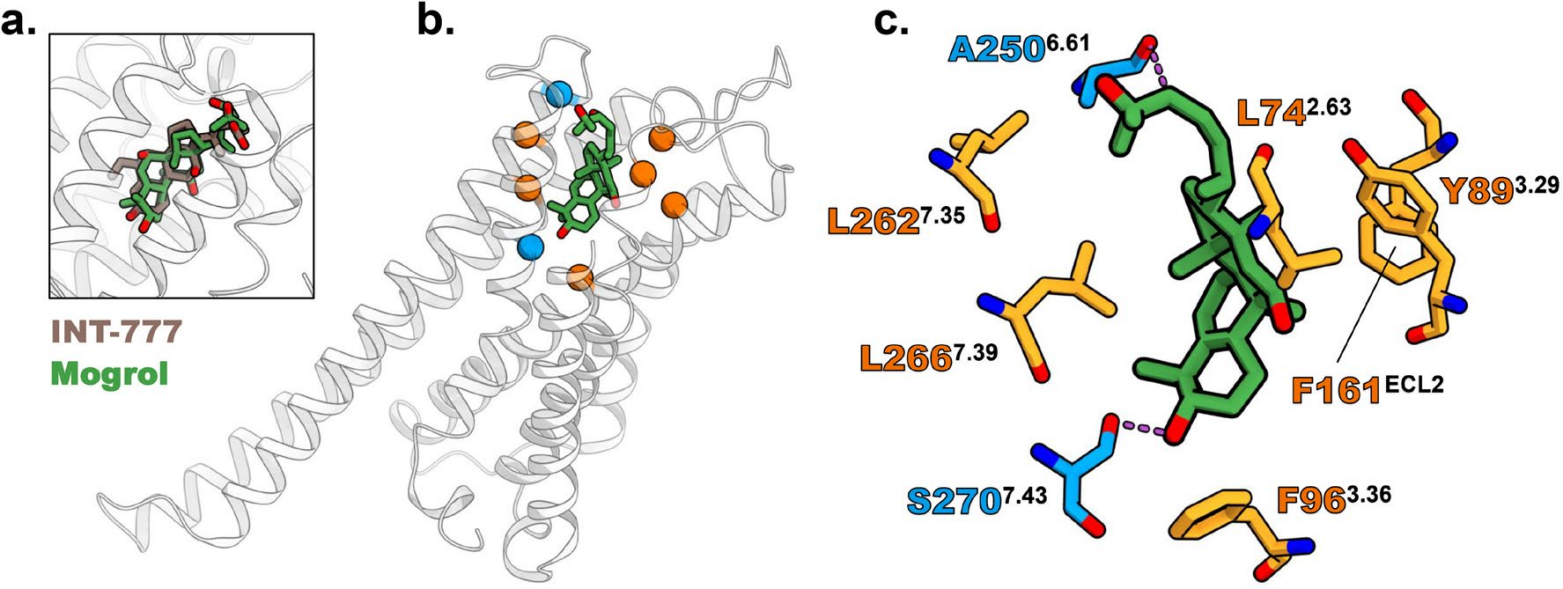
Simulated docking of mogrol to TGR5. (**a**–**c**) Docking results are visually represented as ribbons, sticks, and spheres using CueMol ver. 2.2.3.443 (http://www.cuemol.org/). Grey ribbons represent the TGR5 structure (PDB code: 7CFN). Green, brown, orange, and blue sticks indicate mogrol (docking pose 12), INT-777 (PDB code: 7CFN), TGR5 residues involved in vdW interaction with docked mogrol, and TGR5 residues involved in hydrogen bonding to mogrol, respectively. Orange and blue spheres demarcate TGR5 locations involved in vdW interaction and hydrogen bonding with mogrol, respectively. (**a**) Superposition of mogrol in its 12th docking pose and INT-777 in the cryo-EM structure. (**b**) Mapping of interaction sites detected by Protein–Ligand Interaction Profiler onto the TGR5 structure. (**c**) Details of predicted mogrol–TGR5 interactions. Hydrogen bonds are shown as dashed magenta lines. PDB, Protein Data Bank; TGR5, G-protein-coupled bile acid receptor 1/Takeda G-protein receptor 5.

To identify TGR5 residues involved in mogrol binding, we computationally docked mogrol with TGR5 from the semisynthetic bile acid INT-777-bound TGR5 structure^8^ using GNINA. In 20 docking poses, GNINA pose scores ranged from 0.806 to 0.584. Vina scores ranged from − 10.8 to − 8.77 kcal mol^−1^. The previously reported cryo-EM structure (PDB code: 7CFN) shows that TGR5-bound INT-777 inserts its tetracyclic moiety into the receptor interior^8^. Based on the structural similarity between mogrol and INT-777, we focused on the 12th docking pose (GNINA pose score: 0.64; Vina score: − 9.52 kcal mol^−1^) in which the position of the tetracyclic moiety is close to that of INT-777 in the cryo-EM structure (Fig. 3a). In the focused pose, six residues (L74^2.63^, Y89^3.29^, F96^3.36^, F161^ECL2^, L262^7.35^, and L266^7.39^) were involved in van der Waals (vdW) interactions with mogrol, and the side chain of S270^7.43^ and the main chain of A250^6.61^ were involved in hydrogen bonds to mogrol (Fig. 3b and c).

**Figure 4:**
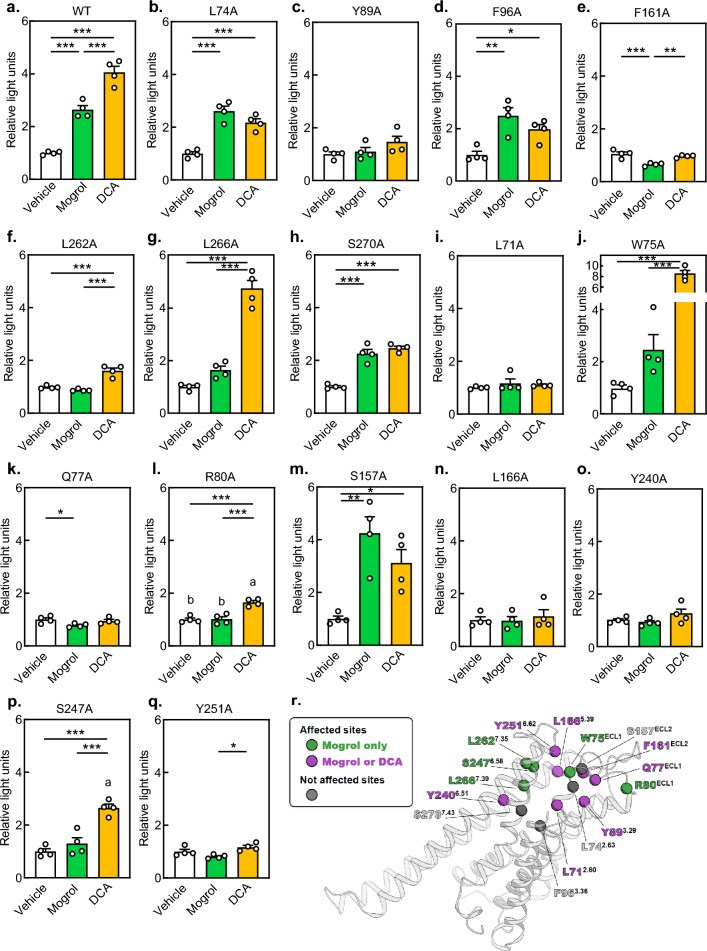
Identification of amino acid residues in TGR5 that are key to activation by mogrol using single amino acid substitutions in the receptor. (**a**–**q**) HEK293FT cells were transiently transfected with pcDNA3.2-wild-type or mutant TGR5 expression vectors, p4xCRE-TATA-Luc2P firefly luciferase reporter vector, and pGL4.74[*hRluc*/TK] *Renilla* luciferase reporter vectors for 24 h. Cells were then stimulated with 50 μM mogrol or 10 μM sodium deoxycholate for 4 h, and luciferase reporter activities were determined. Data are expressed as means ± SEM (n = 4). Asterisks indicate statistically significant differences compared with relevant controls (one-way analysis of variance with Tukey–Kramer’s post hoc tests, *p < 0.05, **p < 0.01, ***p < 0.001). (r) The presumed binding site of TGR5 for mogrol is indicated by color. TGR5, G-protein-coupled bile acid receptor 1/Takeda G-protein receptor 5.

We made vectors to express each mutation to Ala of the amino acids in TGR5 predicted to be important for mogrol binding. A luciferase reporter assay showed that L262A^7.35^ and L266A^7.39^ mutants were not activated by mogrol, but were activated by DCA, whereas Y89A^3.29^ and F161A^ECL2^ mutants were not activated by mogrol or DCA (Fig. 4a–h). The loss of activity in L266A is likely to be mogrol selective. Previous studies have revealed that Q77^ECL1^, R80^ECL1^, and Y89^3.29^ of TGR5 are binding sites for nomilin and obacunone^21^ and L71^2.60^, L74^2.63^, W75^ECL1^, F96^3.36^, S157^ECL2^, F161^ECL2^, L166^5.39^, Y240^6.51^, S247^6.58^, and Y251^6.62^ of TGR5 are important sites for binding to both P395 and INT-777^8^. L74^2.63^, Y89^3.29^, F96^3.36^, and F161^ECL2^ are commonly predicted as agonist binding sites in our docking simulation study (Fig. 3a–c) and the previous reports. Ala mutants, W75A^ECL1^, R80A^ECL1^, and S247A^6.58^ were not activated by mogrol, but were activated by DCA, whereas L71A^2.60^, Q77A^ECL1^, L166A^5.39^, Y240^6.51^, and Y251^6.62^ were not activated by mogrol or DCA (Figs. 4I–Q). Collectively, these results suggest that mogrol binds into the orthosteric binding pocket of TGR5, and W75^ECL1^, R80^ECL1^, S247^6.58^, L262^7.35^, and L266^7.39^ in TGR5 are important for its interaction with mogrol. In addition, L71^2.60^, Q77^ECL1^, Y89^3.29^, F161^ECL2^, L166^5.39^, Y240^6.51^, and Y251^6.62^ appear to be crucial for its interaction with mogrol, but we cannot fully rule out the possibility that these mutations disrupt receptor structure. Identified putative binding site of TGR5 for mogrol was shown in Fig. 4r.

### Mogrol increased insulin secretion by activating TGR5 in β-cells

**Figure 5:**
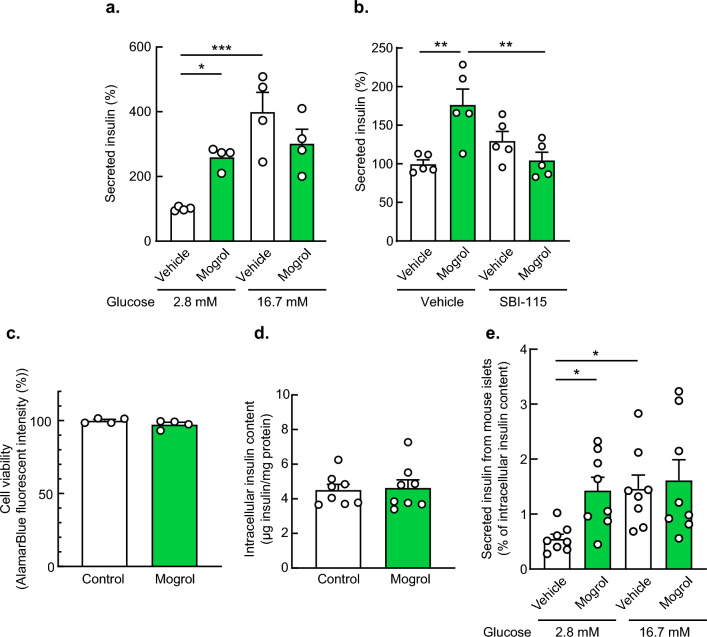
Mogrol activated insulin secretion in INS-1 β-cells and isolated mouse islets. (**a** and **b**) Insulin secretion from INS-1 cells in the presence of 50 μM mogrol and/or 10 μM SBI-115 under low (2.8 mM; **a** and **b**) and high (16.7 mM; **a**) glucose conditions. Amounts of extracellular insulin were determined by ELISA. (**c** and **d**) Cell viability or intracellular insulin content after incubation with 50 μM mogrol for 24 h. (**e**) Insulin secretion from the isolated mouse islets in the presence of 50 μM mogrol under low (2.8 mM) and high (16.7 mM) glucose conditions. Amounts of extracellular and intracellular insulin were determined using ELISA, and the ratio of secreted to intracellular insulin was calculated. Data are expressed as means ± SEM (n = 4–5, for insulin secretion assay in INS-1 cells; n = 4, for cell viability assay in INS-1 cells; n = 8, for measurement of insulin content in INS-1 cells, n = 8, for insulin secretion assay in isolated mouse islets). Asterisks indicate statistically significant differences compared with relevant controls (two-way analysis of variance with Bonferroni’s post hoc tests (**a**, **b**, and **e**) or Student’s *t*-tests (c and d), *p < 0.05, **p < 0.01, ***p < 0.001). TGR5, G-protein-coupled bile acid receptor 1/Takeda G-protein receptor 5.

Activation of TGR5 promotes insulin secretion by pancreatic β-cells^16^. We thus examined the effects of mogrol on insulin secretion by INS-1 β-cells. INS-1 cells were stimulated by mogrol for 1 h, and secreted insulin was determined by ELISA. As shown in Fig. 5a, mogrol significantly stimulated insulin secretion under low-glucose, but not high-glucose, conditions. The enhancement was inhibited by the TGR5 inhibitor SBI-115. (Fig. 5b). Cell viability and intracellular insulin content were not affected by the tested concentrations of mogrol (Fig. 5c and d). In addition, the stimulatory effects of mogrol on insulin secretion under low-glucose conditions were observed in the isolated mouse islets (Fig. 5e).

### Mogrol increased plasma insulin concentrations and alleviated hyperglycemia in diabetic KKAy mice

**Figure 6:**
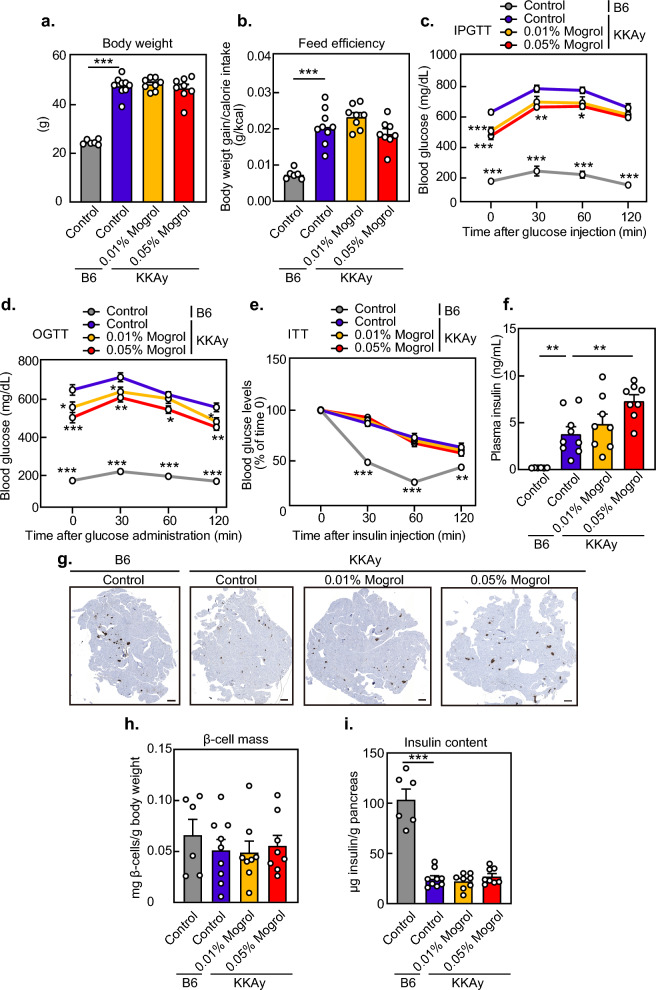
Dietary mogrol increased plasma insulin concentrations and alleviated glucose intolerance in KKAy diabetic mice. KKAy mice, at 5 weeks of age, were fed a high-fat diet for 5 weeks. Two groups received a mogrol supplemented diet (0.01% or 0.05%). C57BL/6 J (B6) mice fed the standard diet were used as normal controls. (**a**) Body weight; (**b**) feed efficiency (i.e., body weight gain/calorie intake); (**c**) intraperitoneal glucose tolerance test (IPGTT) at 7 weeks of age; (**d**) oral glucose tolerance test (OGTT) at 8 weeks of age; (**e**) insulin tolerance test (ITT) at 7 weeks of age; and (**f**) plasma insulin concentrations. (**g**) Representative images of pancreatic staining for insulin. Scale bar, 500 μm. (**h**) Pancreatic β-cell mass per body weight. (**i**) Pancreatic insulin content. Data are expressed as means ± SEM (n = 6, C57BL/6 J mice; n = 8–9, KKAy mice) and were analyzed by one-way analysis of variance with Dunnett’s post hoc tests. Data from glucose and insulin tolerance tests were analyzed at the same time points. Asterisks indicate statistically significant differences compared with the KKAy diabetic control group (*p < 0.05, **p < 0.01, ***p < 0.001).

Because mogrol stimulates insulin secretion and TGR5 activation has been reported to repress hyperglycemia^34^, we examined the effects of mogrol on blood glucose levels in the KKAy diabetic mouse model. KKAy diabetic mice were divided into three groups (*i.e.*, a vehicle control, 0.01% mogrol, and 0.05% mogrol) and treated under these conditions for 5 weeks. C57BL/6 J mice were used as a non-diabetic control group. Although the body weight of KKAy mice was higher than that of C57BL/6 J mice, mogrol supplementation exerted no effects on body weight or feeding efficiency among KKAy mice (Fig. 6a and b). Liver and plasma lipid levels in KKAy mice were not significantly affected by mogrol intake (Table 1), but plasma triglyceride levels tended to decrease (*p* = 0.08 at 0.01% mogrol group). Both intraperitoneal and oral glucose tolerance tests showed that mogrol intake dose-dependently lowered blood glucose concentrations compared with those of KKAy diabetic control mice, whose blood glucose concentrations were elevated compared to C57BL/6 J non-diabetic control mice (Fig. 6c and d). Mogrol intake did not affect insulin sensitivity in KKAy mice (Fig. 6e), but did dose-dependently increase plasma insulin concentrations (Fig. 6f). Pancreatic β-cell mass (Fig. 6g and h) and pancreatic insulin protein levels (Fig. 6i) were not affected by the administration of mogrol.

**Table 1:** Biochemical parameter in the liver and plasma.

		C57BL/6 J	KKAy		
		Control	Control	0.01% Mogrol	0.05% Mogrol
Liver	TG (mg/g liver)	10.4 ± 1.4***	51.7 ± 9.6	48.9 ± 9.1	51.2 ± 9.6
	T-Cho (mg/g liver)	0.27 ± 0.08***	2.54 ± 0.30	2.51 ± 0.18	3.14 ± 0.46
Plasma	TG (mg/dL)	28.9 ± 3.0***	332.7 ± 23.0	281.0 ± 13.4	266.8 ± 30.8
	T-Cho (mg/dL)	94.2 ± 4.2***	299.8 ± 8.7	267.8 ± 13.6	291.2 ± 9.9
	FFA (mEq/L)	0.40 ± 0.03***	1.07 ± 0.09	0.89 ± 0.04	1.07 ± 0.10

Data are expressed as means ± SEM. Statistical differences (*p < 0.05, **p < 0.01, and ***p < 0.001) were determined by comparing KKAy control group (n = 6, C57BL/6 J; n = 8–9, KKAy) with one-way analysis of variance and Dunnett’s post hoc tests. The liver and plasma and were analyzed after dissection. FFA, free fatty acid, T-Cho, total cholesterol; TG, triglyceride.

## Discussion

Recent studies have revealed the physiological effects of mogrol, including as anti-inflammatory and anti-osteoporotic activities in mice^3–6^, but to date, a molecular target of mogrol has not been elucidated. In this study, we showed that mogrol selectively activates TGR5, while other bile acid receptors, including FXR, VDR, and CHRM3, do not^12^. Docking simulation analysis and evaluation of activation using TGR5 mutants indicate that mogrol acts as an agonist for TGR5. Furthermore, we have demonstrated that mogrol functions as an insulin secretagogue by activating TGR5 in pancreatic β-cells and alleviates hyperglycemia in mice.

Mogrol, but not mogrosides, activated TGR5. Activation of TGR5 by mogrol but not by DCA was abolished in variants with the alanine scanning mutations W75A^ECL1^, R80A^ECL1^, S247A^6.58^, L262A^7.35^, and L266A^7.39^. In contrast, activation of TGR5 by mogrol and DCA was abolished by the mutations L71A^2.60^, Q77A^ECL1^, Y89A^3.29^, F161A^ECL2^, L166A^5.39^, Y240^6.51^, and Y251A^6.62^. Our results indicating that mutations L71^2.60^, Y89^3.29^, F161^ECL2^, L166^5.39^, Y240^6.51^, S247^6.58^, Y251A^6.62^, L262^7.35^, and L266^7.39^ reduce mogrol potency were similar to results obtained with INT-777^8^. In addition, our observation that Y89A^3.29^ in TGR5 reduced sensitivity to a bile acid was consistent with a previous study^35^, whereas decreased DCA sensitivity was ameliorated with the S270A^7.43^ mutation relative to the Y89A^3.29^ mutation. W75^ECL1^ acts as an active site at lid^36^, and mutation at this site can selectively increase DCA potency or decrease the potency of mogrol and P395^8^ but does not affect 23H or litocholic acid^36^. Sasaki et al.^21^ have shown that limonoid compounds (the most abundant triterpenoids in citrus fruits) nomilin and obacunone activate TGR5, whereas in the triple mutants Q77R^ECL1^/R80Q^ECL1^/Y89H^3.29^ and Q77A^ECL1^/R80A^ECL1^/Y89A^3.29^ activation by nomilin and/or obacunone, but not taurolithocholic acid, was abolished^21^. These results suggest that mutations in Q77^ECL1^, R80^ECL1^, and Y89^3.29^ exert a negligible effect on overall structure of TGR5. F96A^3.36^ compromised the potency of the TGR5 agonist 23H but not that of litocholic acid^36^. Our result with the F96A mutant was similar to the results with litocholic acid. Overall, these results indicate that mogrol binds directly to and acts as an agonist of TGR5.

TGR5 was not activated by mogrosides, glycosides of mogrol, whereas the glycosides of quinovic acid^18^ and glycyrrhizic acid (a glycoside of glycyrrhetinic acid)^20^ can activate this receptor. The hypoglycemic effect of ginsenoside Ro, a glycoside of the TGR5 agonist oleanolic acid, was attenuated in TGR5-deficient mice^37^, whereas it remains unclear whether ginsenoside Ro is itself without deglycosidation or has a direct effect on β-cells. Mogrol-stimulated insulin secretion was inhibited by the TGR5 inhibitor, suggesting that mogrol stimulates insulin secretion by activating TGR5. Our results support the hypothesis that TGR5 activation stimulates insulin secretion in pancreatic β-cells^16^. Although CHRM3 is expressed in β-cells and contributes to insulin secretion^38^, mogrol did not affect CHRM3 activation. To our knowledge, the ability of terpenoids and related compounds to modulate insulin secretion by activating TGR5 has not previously been experimentally demonstrated.

Mogrol increased the insulin secretion under low-glucose conditions in INS-1 cells and isolated mouse islets. Kumar et al.^16^ showed that TGR5 couples with Gαs, but not Gαq, Gαi, and Gα_12/13_, in MIN6 β-cells and that the activation of TGR5 by oleanolic acid (10 μM), INT-777, and lithocholic acid enhances insulin secretion both under low- and high-glucose concentrations in MIN6 and human β-cells. In addition, the intracellular calcium levels increased at low-glucose concentrations. Although overdosage of insulin secretagogues should be noted in relation to hypoglycemia, to our knowledge, the occurrence of hypoglycemia by TGR agonists has not been reported. Tauroursodeoxycholic acid and oleanolic acid (1 μM) increases the glucose-stimulated insulin secretion from the mouse or human islets under high-glucose, but not low-glucose, concentrations^15,39^. These results suggest that different ligands may alter the binding mode of the TGR5 receptor and activate different signals, particularly intracellular calcium signaling. Different concentrations of compound could affect cellular signaling in complex ways. The reduced insulinotropic effect of mogrol under high-glucose concentrations may be due to its AMPK activity-promoting effect^3,7,40^ as AMPK activation has a negative impact on insulin secretion^41^, although this remains controversial^42^.

In contrast to some TGR5 agonists^37,43,44^, mogrol did not affect the body weight or insulin sensitivity in mice. Muscle-specific TGR5 transgenic mice exhibit increased glucose tolerance and a slight improvement in insulin sensitivity (*P* = 0.066), without any change in their body weight^45^. Therefore, the effects of muscle TGR5 activation on insulin sensitivity may be limited. The bioavailability, tissue distribution, and tissue accumulation of compounds may have caused these differences.

Dietary supplementation (0.4%) with crude extract of *Siraitia grosvenorii* (SG-ex) prevented impaired insulin secretion and glucose intolerance in Goto-Kakizaki rats, presumably due to decreased oxidative stress in the pancreas^46^. By contrast, a single administration of a mogroside-rich extract of *Siraitia grosvenorii* (SG-gly) can alleviate a rise in blood glucose concentrations after maltose but not glucose loading in rats^47^. SG-gly contents in SG-ex are low. Thus, anti-oxidative components other than SG-gly in SG-ex seem to be responsible for the observed anti-hyperglycemic effects. Therefore, compounds from *Siraitia grosvenorii* have several targets by which they may ameliorate hyperglycemia (*i.e.*, SG-gly, maltase inhibitory activity; SG-ex, anti-oxidative activity in the pancreas; mogrol, insulin secretory activity).

In this study, we have demonstrated that TGR5 is a target of mogrol and have identified several amino acids in TGR5 that are key to mogrol binding. Mogrol activated CRE-mediated transcriptional activation in TGR5-expressing cells. By contrast, in 3T3-L1 preadipocytes, mogrol suppresses CRE-mediated CREB activity^7^. This inconsistency may be due to the existence of mogrol targets other than TGR5. In the present study, we showed that mogrol activated TGR5 and insulin release from pancreatic β-cells, alleviating hyperglycemia at least partly in diabetic KKAy mice. Further studies to identify other mogrol targets could uncover different mechanisms underlying mogrol mediated physiological effects. A limitation of this study is that the TGR5-mediated function of mogrol lacks in vivo experiment. Further mechanistic studies of mogrol using gene knockout animals are warranted. Mogrosides are natural sweeteners that can be used as sugar alternatives. After ingestion, mogrol is produced by the deglycosylation of mogrosides in the intestinal tract and subsequently absorbed by the body^1,2^. Therefore, mogroside intake, in addition to direct mogrol supplementation, may contribute to the prevention of T2DM by exerting effects other than the reduction of sugar intake.

## Methods

### Materials

Mogrol and mogrosides were prepared as described previously^7^. Their structures are shown in Fig. 1. These compounds were purified to at least 96% via HPLC and dissolved in DMSO.

### Plasmid construction

To construct the human FXR expression vector p3xFlag-FXR and the human vitamin D receptor (VDR) expression vector pcDNA3.1-VDR, nested PCR reactions were performed using PrimeSTAR HS DNA polymerase and Gflex DNA polymerase (Takara Bio, Shiga, Japan) using cDNA reverse-transcribed from total RNA of human hepatoma HepG2 cells. Primers used in the 1st and 2nd PCR reactions for FXR cloning were as follows: 1st sense: 5′- TCCTCAAGATGAAACTTCAGACAC-3′; 1st antisense: 5′-ATGCAGGATTCCCTGGAGCCTTT-3′; 2nd sense: 5′-GGCGGCCGCCACCATGGGATCAAAAATGAATCTC-3′; 2nd antisense: 5′-GGGATCCTCACTGCACGTCCCAGATTTC-3′. Primers used in the 1st and 2nd PCR reactions for VDR cloning were as follows: 1st sense: 5′-CAGAAGCCTTTGGGTCTGAAGTGTC-3′; 1st antisense: 5′-CAACATCAGTCAGCAGCCACTTAGG-3′; 2nd sense: 5′-GGCGGCCGCCACCATGGAGGCAATGGCGGCCAGC-3′; 2nd antisense: 5′-GGGATCCTCAGGAGATCTCATTGCCAAAC-3′. After cloning cDNA sequences into the pCR2.1-TOPO-TA vector (Thermo Scientific, Waltham, MA, USA), *Not* I- and *BamH* I-digested fragments encoding FXR and VDR were inserted into the corresponding sites in p3xFLAG-CMV (Sigma-Aldrich, St. Louis, MO, USA) and pcDNA3.1 (Thermo Fisher Scientific), respectively. To construct luciferase reporter vectors for FXR (p4xFXRE-TATA-Luc2P), VDR (p2xVDRE-TATA-Luc2P), and NFAT (p9xNFAT-RE-TATA-Luc2P), annealed oligonucleotides containing four tandem copies of a consensus FXR-responsive IR1 motif^48^ (sense: 5′-ATAGCACAAGAGGTCATTGACCTTGTCCACAAGAGGTCATTGACCTTGTCCACAAGAGGTCATTGACCTTGTCCACAAGAGGTCATTGACCTTGTCCA-3′, and antisense: 5′-GATCTGGACAAGGTCAATGACCTCTTGTGGACAAGGTCAATGACCTCTTGTGGACAAGGTCAATGACCTCTTGTGGACAAGGTCAATGACCTCTTGTG-3′), annealed oligonucleotides containing two tandem copies of mouse osteopontin VDR response element^49^ (sense: 5′-CTAGCACAAGGTTCACGAGGTTCACGTCTACAAGGTTCACGAGGTTCACGTCTA-3′, and antisense: 5′-GATCTAGACGTGAACCTCGTGAACCTTGTAGACGTGAACCTCGTGAACCTTGTG-3′), and annealed oligonucleotides containing nine tandem copies of an IL-8 NFAT response element^50^ (sense: 5′-CTAGCTTGAGGAATTTCCATTGAGGAATTTCCATTGAGGAATTTCCATTGAGGAATTTCCATTGAGGAATTTCCATTGAGGAATTTCCATTGAGGAATTTCCATTGAGGAATTTCCATTGAGGAATTTCCATTA-3′, and antisense: 5′-GATCTAATGGAAATTCCTCAATGGAAATTCCTCAATGGAAATTCCTCAATGGAAATTCCTCAATGGAAATTCCTCAATGGAAATTCCTCAATGGAAATTCCTCAATGGAAATTCCTCAATGGAAATTCCTCAAG-3′) and adenovirus E1b TATA box sequence (sense: 5′-AGCTTAGGGTATATAATGA-3′ and antisense: 5′-TCATTATATACCCTAAGCT-3′) were sequentially inserted into the vector pGL4.11 (Promega, Madison, WI, USA), respectively. Mutant TGR5 expression vectors were constructed by site-directed mutagenesis using Tks Gflex DNA polymerase (Takara Bio) with specific primers (Table S1) and pcDNA3.2-TGR5^24^ as a template. General residue numbering has been used, with sequential numbers retrievable from the GPCRdb^51^.

### Cell culture

HEK293FT cells (Thermo Fisher Scientific) and HepG2 cells (RIKEN Cell Bank, Tsukuba, Japan) were cultured in Dulbecco's Modified Eagle Medium supplemented with 10% fetal bovine serum, 100 U/ml sodium penicillin G, and 100 μg/ml streptomycin sulfate. INS-1 rat pancreatic β-cells^52^ were cultured in RPMI 1640 medium supplemented with 10% fetal bovine serum, 10 mM HEPES, 1 mM sodium pyruvate, 50 μM 2-mercaptoethanol, 100 U/ml sodium penicillin G, and 100 μg/ml streptomycin sulfate. Cells were maintained at 37 °C in an atmosphere containing 5% CO_2_ and 95% air.

### Luciferase reporter assay

HEK293FT cells were seeded in Dulbecco’s Modified Eagle Medium containing 10% fetal bovine serum without antibiotics. After overnight culture, cells were transfected with a receptor expression vector (pcDNA3.2-TGR5^24^, p3xFlag-FXR, pcDNA3.2-VDR, and pcDNA3.2-CHRM3), a firefly luciferase reporter vector (p4xCRE-TATA-Luc2P^53^, p4xFXRE-TATA-Luc2P, p2xVDRE-TATA-Luc2P, and p9xNFAT-RE-TATA-Luc2P), and a *Renilla* luciferase reporter vector (pGL4.74[*hRluc*/TK] (Promega)) with Opti-MEM (Thermo Fisher Scientific) and PEI MAX (Polysciences Inc, Warrington, PA, USA) for 24 h. The cells were subsequently incubated in the presence of mogrol for 4 h. Luciferase reporter activity was determined as described previously^54^. Data are expressed as relative light units.

### Cell viability assay

INS-1 cells grown in a 48-well plate were cultured with 50 μM mogrol for 24 h, followed by incubation with 5% alamarBlue (Bio-Rad, Hercules, CA, USA) for 4 h under light shielding. Fluorescence was measured at excitation and emission wavelengths of 544 and 590 nm, respectively, using Fluoroscan Ascent FL (Labsystems, Helsinki, Finland).

### Insulin secretion assay and measurement of insulin levels in INS-1 cells

The amounts of insulin secreted by INS-1 cells were determined as previously described^53^. Briefly, INS-1 cells seeded in a 48-well plate were incubated with the Krebs–Ringer bicarbonate buffer containing 0.1% bovine serum albumin and glucose (2.8 or 16.7 mM) in the presence or absence of 50 μM mogrol for 1 h. Cells were pretreated with the TGR5 inhibitor, SBI-115, for 40 min, followed by mogrol treatment. INS-1 cells grown in a 48-well plate were cultured with 50 μM mogrol for 24 h. Cell lysates were prepared using a lysis buffer (50 mM Tris–HCl, 150 mM NaCl, 0.5% NP-40, and 2 mM EDTA). The amounts of secreted insulin and intracellular insulin were determined using sandwich ELISA as previously described^53^. Bovine insulin was used as the standard.

### Insulin secretion assay using isolated mouse islets

Pancreatic islets were isolated from 8–9-week-old ICR mice (Kiwa Laboratory Animals, Wakayama, Japan) as reported previously^53^, with minor modification. Briefly, after collagenase digestion, the pancreas was fractionated via Ficoll-diatrizoate density gradient centrifugation. Mouse islets (3–4 islets/tube) were incubated with the Krebs–Ringer bicarbonate buffer containing 0.1% bovine serum albumin with a basal (2.8 mM) or stimulatory (16.7 mM) level of glucose for 1 h in the presence of mogrol. Secreted and intracellular insulin concentrations were measured using ELISA, as previously described^53^. Finally, the ratio of secreted to intracellular insulin was calculated.

### Structure-based docking simulation

Coordinates from a structure of human TGR5–G_s_ complex with bound bile acid derivative INT-777, solved by cryo-electron microscopy (cryo-EM) (Protein Data Bank [PDB] code: 7CFN)^8^ were used for our docking studies examining TGR5–mogrol binding. A three-dimensional structure of mogrol was obtained from PubChem (CID: 14,525,327). Hydrogen atoms and missing heavy atoms were added to the experimental TGR5 structure using Protein Repair and Analysis Server^55^. In silico docking of the mogrol molecule with the TGR5 structure was performed using GNINA^56^ version 1.0.2 (derivative of SMINA^57^ and AutoDock Vina^58,59^ that supports convolutional neural network scoring). The docking box in the simulation was automatically set up with reference to the INT-777 coordinates in 7CFN. During docking simulations, the rotational bands of the small molecule were explicitly considered to be flexible, and the protein was treated as a rigid body. TGR5–mogrol interactions in the docking results were analyzed and detected using Protein–Ligand Interaction Profiler^60,61^ version 2.2.2.

### Animals

Male 4-week-old C57BL/6 J and KKAy mice were obtained from CLEA Japan (Tokyo, Japan) and individually housed (cage size: 136 × 208 × 115 mm; bedding material: clean chip, CLEA Japan). Additionally, male 8-week-old ICR mice were obtained from Kiwa Laboratory Animals and housed in a group (cage size: 225 × 338 × 140 mm; bedding material: clean chip) under conventional conditions with controlled temperatures (23 ± 3 °C), a 12/12-h light/dark cycle (light period starting at 08:00 AM), and ad libitum access to standard food (CE-2, CLEA Japan) and water. After acclimation for one week, C57BL/6 J mice (the non-diabetic control group, n = 6, 20.4 ± 0.2 g) continued to receive the standard diet for 5 weeks, while KKAy diabetic mice were divided into three groups (n = 8–9, diabetic control group, 28.4 ± 0.2 g; 0.01% mogrol group, 28.7 ± 0.4 g; 0.05% mogrol group, 28.5 ± 0.7 g), with each group having a similar body weight distribution and fed a high-fat diet (HFD)^62^ for 5 weeks. The KKAy diabetic control group was fed HFD alone, and the other groups were fed HFD containing 0.01 or 0.05% (w/w) mogrol. For the intraperitoneal glucose tolerance test (IPGTT), oral glucose tolerance test (OGTT), and insulin tolerance test (ITT), the mice fasted for 6 h (from 09:00 AM), then 2 g/kg glucose or 1 U bovine insulin were intraperitoneally or orally administered. Blood was collected from the tail vein, and glucose levels were measured using a Stat Strip Express Glucose/Ketone meter (Nova Biomedical, Waltham, MA, USA). These tolerance tests were performed in animal care room. After 4 h of fasting (from 09:00 AM), mice were kept unconscious under isoflurane anesthesia (5% for introduction and 2% for maintenance, SN-487, Shinano Seisakusho, Tokyo, Japan) and euthanized by exsanguination from inferior vena cava, and blood and organs were harvested for further analysis. Four groups of mice were treated and dissected alternately. After acclimation for one week, ICR mice (n = 2) were euthanized via cervical dislocation, and the pancreatic islets were isolated. All animal experiments were approved by the Animal Care and Use Committee of the Osaka Metropolitan University (Nos. 22–17 and 23–132) and were performed in compliance with its guidelines and the ARRIVE guidelines.

### Immunohistochemistry and measurement of pancreatic insulin levels

Pancreatic β-cell mass was determined as previously described^63^. Briefly, formalin-fixed paraffin-embedded pancreatic tissue sections were prepared. The sections were mixed with an anti-insulin antibody (D6C4, Hytest, Turku, Finland), Histofine Simple Stain mouse MAX-PO (Nichirei, Tokyo, Japan), and DAB peroxidase substrate kit (Vector Laboratories, Burlingame, CA, USA), followed by counterstaining with hematoxylin. The tissue sections were observed under a light microscope (BZ-X810, Keyence, Osaka, Japan), and the staining intensity was determined using the Image Pro software (ver. 10, Media Cybernetics, Silver Spring, MD, UDA). β-cell mass was calculated as follows: ratio of β-cell area to total pancreatic area × pancreatic weight/body weight. To determine the pancreatic insulin content, the pancreas were homogenized in acid–ethanol (1.5% HCl in 70% ethanol). After neutralization with 1 M Tris–HCl (pH 7.5), insulin concentrations were determined using ELISA, as previously described^53^, and normalized to the pancreatic protein content.

### Measurement of insulin, triglyceride, cholesterol, and free fatty acid levels

Plasma insulin levels were determined using an Ultra Sensitive Mouse Insulin ELISA Kit (Morinaga Institute of Biological Science Inc., Kanagawa, Japan). Lipids were extracted from the liver by the Folch’s method. Liver and plasma triglyceride, total cholesterol, and free fatty acid (FFA) levels were determined with a triglyceride E-test (Fujifilm Wako, Osaka, Japan), a cholesterol E-test (Fujifilm Wako), and a NEFA C-test (Fujifilm Wako), respectively. Measurements were performed single or duplicate.

### Statistical analyses

Data were analyzed by two-tailed Student’s* t*-test or one-way analysis of variance with Tukey–Kramer’s or Dunnett’s post hoc tests using JMP statistical software version 8.0.1 (SAS Institute, Cary, NC, USA). Two-way analysis of variance with Bonferroni’s post hoc test was performed using GraphPad Prism statistical software version 5 (GraphPad Software Inc., San Diego, CA, USA). Data are displayed as means ± SEM, and a *p*-value < 0.05 was considered to indicate a statistically significant difference between groups.

### Supplementary Information

Supplementary Information.

## Data Availability

The data generated during the current study are available from the corresponding author (N.H.) upon reasonable request.
