# Supplementary material for: Mogrol stimulates G-protein-coupled bile acid receptor 1 (GPBAR1/TGR5) and insulin secretion from pancreatic β-cells and alleviates hyperglycemia in mice
Source: Sci Rep. 2024 Feb 8;14:3244. doi: 10.1038/s41598-024-53380-x (PMC10853268; doi:10.1038/s41598-024-53380-x)
Supplement: Supplementary file 1 — Supplementary Information. [file 41598_2024_53380_MOESM1_ESM.docx]

**Mogrol stimulates G-protein-coupled bile acid receptor 1 (GPBAR1/TGR5) and insulin secretion from pancreatic β-cells and suppresses hyperglycemia in mice**

Chisato Tanaka, Naoki Harada, Yoshiaki Teraoka, Hiroki Urushizaki, Yoh Shinmori, Teruaki Onishi, Yusuke Yotsumoto, Yuta Ito, Tomoya Kitakaze, Takashi Inui_,_ Yuji Murata, Hiroshi Inui, and Ryoichi Yamaji.

**Supplementary Table 1.** Primers for construction of mutant TGR5 expression vectors

| Mutation | Sequences |
| --- | --- |
| TGR5-L71A | Fw: 5′-CTGGCATTGCCCACAGCGCCAGGGCTGTGG-3′ |
|  | Rev: 5′-CCACAGCCCTGGCGCTGTGGGCAATGCCAG-3′ |
| TGR5-L74A | Fw: 5′-CCACATTGCCAGGGGCGTGGAACCAGAGTCG-3′ |
|  | Rev: 5′-CGACTCTGGTTCCACGCCCCTGGCAATGTGG-3′ |
| TGR5-W75A | Fw: 5′-CATTGCCAGGGCTGGCGAACCAGAGTCGCCG-3′ |
|  | Rev: 5′-CGGCGACTCTGGTTCGCCAGCCCTGGCAATG-3′ |
| TGR5-Q77A | Fw: 5′-CAGGGCTGTGGAACGCGAGTCGCCGGGGTTAC-3′ |
|  | Rev: 5′-GTAACCCCGGCGACTCGCGTTCCACAGCCCTG-3′ |
| TGR5-R80A | Fw: 5′-GGAACCAGAGTCGCGCGGGTTACTGGTCCTG-3′ |
|  | Rev: 5′-CAGGACCAGTAACCCGCGCGACTCTGGTTCC-3′ |
| TGR5-Y89A | Fw: 5′-CCTGCCTCCTCGTCGCCTTGGCTCCCAAC-3′ |
|  | Rev: 5′-GTTGGGAGCCAAGGCGACGAGGAGGCAGG-3′ |
| TGR5-F96A | Fw: 5′-GCTCCCAACTTCTCCGCCCTCTCCCTGCTTGC-3′ |
|  | Rev: 5′-GCAAGCAGGGAGAGGGCGGAGAAGTTGGGAGC-3′ |
| TGR5-S157A | Fw: 5′-GCCAACTGCAGCGCCCAGGCTATCTTC-3′ |
|  | Rev: 5′-GAAGATAGCCTGGGCGCTGCAGTTGGC-3′ |
| TGR5-F161A | Fw: 5′-GCTCCCAGGCTATCGCCCCAGCCCCCTACC-3′ |
|  | Rev: 5′-GGTAGGGGGCTGGGGCGATAGCCTGGGAGC-3′ |
| TGR5-L166A | Fw: 5′-CCCAGCCCCCTACGCGTACCTCGAAGTC-3′ |
|  | Rev: 5′-GACTTCGAGGTACGCGTAGGGGGCTGGG-3′ |
| TGR5-Y240A | Fw: 5′-GTGCTGGGGGCCCGCCGTGGCCACACTGC-3′ |
|  | Rev: 5′-GCAGTGTGGCCACGGCGGGCCCCCAGCAC-3′ |
| TGR5-S247A | Fw: 5′-CACACTGCTCCTCGCAGTCCTGGCCTATG-3′ |
|  | Rev: 5′-CATAGGCCAGGACTGCGAGGAGCAGTGTG-3′ |
| TGR5-Y251A | Fw: 5′-CTCTCAGTCCTGGCCGCTGAGCAGCGCCCG-3′ |
|  | Rev: 5′-CGGGCGCTGCTCAGCGGCCAGGACTGAGAG-3′ |
| TGR5-L262A | Fw: 5′-GGGGCCTGGGACAGCGTTGTCCCTCCTCTC-3′ |
|  | Rev: 5′-GAGAGGAGGGACAACGCTGTCCCAGGCCCC-3′ |
| TGR5-L266A | Fw: 5′-CACTGTTGTCCCTCGCCTCCCTAGGAAGTG-3′ |
|  | Rev: 5′-CACTTCCTAGGGAGGCGAGGGACAACAGTG-3′ |
| TGR5-S270A | Fw: 5′-CTCCTCTCCCTAGGAGCTGCCAGTGCAGCGG-3′ |
|  | Rev: 5′-CCGCTGCACTGGCAGCTCCTAGGGAGAGGAG-3′ |
